# The impact of the environment on health by country: a meta-synthesis

**DOI:** 10.1186/1476-069X-7-7

**Published:** 2008-02-25

**Authors:** Annette Prüss-Üstün, Sophie Bonjour, Carlos Corvalán

**Affiliations:** 1 World Health Organization, Department of Public Health and Environment, 20 Avenue Appia, 1211 Geneva 27, Switzerland

## Abstract

**Background:**

Health gains that environmental interventions could achieve are main questions when choosing environmental health action to prevent disease. The World Health Organization has recently released profiles of environmental burden of disease for 192 countries.

**Methods:**

These country profiles provide an estimate of the health impacts from the three major risk factors 'unsafe water, sanitation & hygiene', 'indoor air pollution from solid fuel use' and 'outdoor air pollution'. The profiles also provide an estimate of preventable health impacts by the environment as a whole. While the estimates for the three risk factors are based on country exposures, the estimates of health gains for total environmental improvements are based on a review of the literature supplemented by expert opinion and combined with country health statistics.

**Results:**

Between 13% and 37% of the countries' disease burden could be prevented by environmental improvements, resulting globally in about 13 million deaths per year. It is estimated that about four million of these could be prevented by improving water, sanitation and hygiene, and indoor and outdoor air alone. The number of environmental DALYs per 1000 capita per year ranges between 14 and 316 according to the country. An analysis by disease group points to main preventions opportunities for each country.

**Conclusion:**

Notwithstanding the uncertainties in their calculation, these estimates provide an overview of opportunities for prevention through healthier environments. The estimates show that for similar national incomes, the environmental burden of disease can typically vary by a factor five. This analysis also shows that safer water, sanitation and hygiene, and safer fuels for cooking could significantly reduce child mortality, namely by more than 25% in 20 of the lowest income countries.

## Background

"What are the possible health gains due to environmental interventions?" and "How much disease burden could be averted by implementing them?". These are main questions for decision-making towards public health action. Quantifying the disease burden caused by the environment has however been difficult given the relative lack of evidence.

Nevertheless, thanks to the development of new tools in epidemiologic analysis and of methods to estimate population health impacts, several estimates of environmental burden of disease have been developed. While the health impacts from, for example, outdoor air pollution have been developed since about two decades, more recent comprehensive and comparative analyses and methods include: (a) a comparative risk assessment of 26 risk factors, six of which are environmental [1]; (b) estimates of the global impact of the environment on health [2,3]; and (c) the Environmental Burden of Disease series [4] providing country guidance to estimate the burden from selected risks (based on the same methodology as (a) but covering additional risk factors). At national level, several similar analyses have been developed, highlighting an interest for such information [5-10]. Recently, the World Health Organization has developed country profiles of environmental burden of disease.

This article presents the methods and results of the recently published 192 country profiles of environmental burden of disease [11] and discusses their comparison across countries. These are the first country-by-country estimates of the impact of the total environment on health. These profiles can be used as input to a country process of developing a more refined estimate of their health impacts of the environment. They can also be used for intra-country comparisons or serve as preliminary orientation of national decision-makers to set priorities for preventive environmental health action.

## Methods

**Figure 1:**
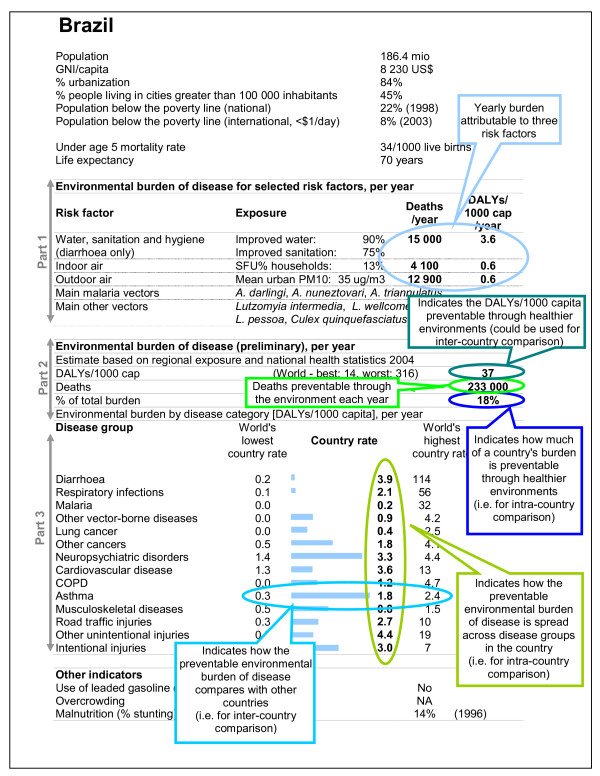
Example of country profile of environmental burden of disease. Overlaid by reading guide for explanatory purposes.

The WHO country profiles on environmental burden of disease (Figure 1) are composed of three parts. Part 1 (a) provides "exposure-based" estimates for three risk factors, i.e. based on globally available country exposures, and for which quantitative methods for disease burden estimation have been published [4]. These three risk factors include 'unsafe water, sanitation and hygiene', 'indoor air pollution from solid fuel use' and 'outdoor air pollution'. Part 1 also includes main malaria and other vectors that are present in the country and cause certain health risks. Part 2 (b) is a preliminary estimate of the total environmental burden of disease for the country, based on a review of the evidence completed by expert opinion. Part 3 (c) presents a breakdown by disease group for the estimate provided in Part 2.

In addition, the country profiles list a number of socio-economic parameters such as the GNI (Gross National Income), population, population living in poverty, urbanization and others in order to better understand the country's situation. Also, selected environmental exposures are presented to provide additional information regarding prevention opportunities [11]. These additional exposures were selected because they are both globally available and relevant for quantification of various health outcomes: lead (e.g. associated with cognitive impairment), malnutrition (associated with most infectious diseases, and water, sanitation and hygiene) and crowding (e.g. associated with the transmission of tuberculosis). The impact of environment-related malnutrition has already been incorporated in the EBD estimates (see [12] for additional details relating to water, sanitation and hygiene in particular). No comprehensive database was, however, available for blood lead levels, and no methods for estimating the disease burden due to crowding had been developed at the time of estimation. Such country-level exposures can be used for refined estimates at country level.

Both exposure-based estimates (Part 1) and evidence reviews completed by expert opinion-based methods (Part 2 and 3) use population attributable fractions, but differ in its estimation method. To obtain the environmental burden of disease, these attributable fractions are then multiplied by the total burden of disease (in deaths or DALYs) of the relevant disease (see [13] or Volume 1 of [4] for additional information on the Global Burden of Disease concept). The population attributable fraction is defined as the proportional reduction in disease or death that would occur if exposure to the risk factor were reduced to zero (see Chapter 1 of [14] or Volume 1 of [4] for references and explanations).

Attributable disease burden as estimated here – based on attributable fractions – is in principle not equivalent to preventable burden. Rather, preventability also depends on the technical, social, economic, psychological and ethical dimensions of a situation [15]. This being said, such burden points to the impact of causes, and the potential for prevention under certain circumstances. To some extent, the preventability of the attributable burden is also influenced by its method of estimation (in particular the counterfactual, or reference value, against which the impact of current exposure burden was estimated, or in the case of expert evaluation the formulation of the question; see sections (a) and (b) below).

While the results are expressed in terms of total deaths or a summary measure of population health, more detailed information is sometimes available (such as by age group or region, provided the underlying epidemiological information was specific enough).

The following sections provide additional details on the methods underlying the Parts 1 to 3 of the country profiles.

### (a) Exposure-based estimates (Part 1)

The exposure to the three risks 'unsafe water, sanitation & hygiene', 'indoor air pollution from solid fuel use' and 'outdoor air pollution' has been assessed or estimated globally, and the databases with country exposures are publicly accessible [16-18]. These data are mainly based on household surveys for water, sanitation and solid fuel use, and measurements for outdoor air pollution. Missing data were modeled. For example, the proportion of households using solid fuels was available for 93 of 181 countries; modelled for 36 countries based on proportion of rural population and gross national income (GNI); and assumed as less than 5% of households using solid fuels for the 52 higher income countries with GNI above US$ 10,500. The diseases taken into account include the following:

• diarrhoea for water, sanitation and hygiene;

• acute lower respiratory infections (in children under 5 years), chronic obstructive pulmonary disease and lung cancer (in adults above 30 years) for solid fuel use; and

• respiratory mortality (in children under 5 years), cardiopulmonary mortality and lung cancer (in adults above 30 years) for outdoor air pollution.

The disease statistics used for this analysis have been compiled by the World Health Organization, by country, gender and age group [19]. The methods used to combine exposure data and disease statistics into attributable disease burden have been published in the environmental burden of disease series [20-22], based on previous global analyses [1,23-25]. Detailed information on the input data and methods can be found in the respective sources, which are all publicly accessible.

The method for obtaining exposure-based estimates can (for the risks studied here) be summarized as combining exposure with risk measures to obtain a population attributable fraction (simplified e.g. from [26,27]):

$$AF=\frac{\sum_{i=1}^{n}Pi(RRi-1)}{\sum_{i=1}^{n}Pi(RRi-1)+1}$$

Where: *i *is the exposure category, *Pi *is the proportion of the population in exposure category *i*, and *RRi *is the relative risk at exposure category *i *compared with the reference level.

The disease burden from water, sanitation and hygiene and indoor air pollution reflects the total attributable burden, in other words the burden that could be avoided if water, sanitation and hygiene, and solid fuel use could be improved to the point of not causing any health impact. The estimates for outdoor air pollution however reflect the disease burden that could be prevented if pollution levels were reduced to WHO guideline values for particulate matter, although it is acknowledged that adverse health effects occur even below this value [28].

The exposure-based assessment has been carried out for only these three risk factors because they were the only ones with both a method for quantified estimation of health impacts and global databases of exposure assessment available. While for some additional risk factors such global quantification may become possible with development of additional methods (e.g. for second-hand smoke, crowding), it is more difficult for risk factors for which population exposure is more cumbersome to assess (such as for ionizing radiation). We therefore proceeded to complete exposure-based assessments with literature review/expert-based estimates (Part 2 and 3).

Results are expressed in premature deaths (as compared to standard life expectancy) and in DALYs (Disability-Adjusted Life Years), a combination of death and disability (Additional file 1 contains mainly deaths, whereas the full country profiles contain both deaths and DALYs for each risk factor). DALYs for a disease are the sum of the years of life lost due to premature mortality (YLL) in the population and the years lost due to disability (YLD) for incident cases of the health condition [29]. DALYs therefore reflect the age of death, which means that deaths occurring mostly at more advanced age produce fewer DALYs than deaths occurring primarily in childhood.

Exposure-based estimates of disease burden should, in principle, not be added up across risk factors, as disease could be prevented by acting on different risks. For example, diarrhoea deaths could be prevented by improving nutritional status, water, sanitation and hygiene, or food safety. To avoid double-counting, one practical approach consists in avoiding to sum up disease burden within one disease category altogether, or otherwise to carefully consider the potential for double counting in the specific cases.

### (b) Preliminary estimate of the total environmental burden of disease for the country (Part 2)

The data presented in the second part of the country profiles represent the disease burden that could be avoided by modifying the environment as a whole. The "modifiable environment" includes pollution of air, water and soil; radiations; noise; occupational risks; the built environment, including housing and road design; land use patterns; agricultural methods and irrigation schemes; man-made changes to the climate and ecosystems, and behaviour related to the environment (such as hand-washing or personal protection). Excluded from the definition are individual choices, such as alcohol and tobacco consumption, drug abuse, diet; natural environments or ecosystems that cannot reasonably be modified (rivers, etc); unemployment (provided that it is not linked to the degradation of the environment); natural biological agents (e.g. pollen); person-to-person transmission that cannot reasonably be prevented by environmental interventions.

The total environmental burden has been estimated on a disease-by-disease approach, rather than by risk factor. For 102 diseases or injuries, the literature has been systematically reviewed in terms of attribution to or preventability by environmental improvements, and completed by a survey of over 100 experts worldwide [2]. In the survey, experts were asked to focus on the modifiable part of the environment, hereby indicating the disease burden that could potentially be prevented (or shifted to health risks that lie outside the environmental area). Experts were selected on the basis of their international expertise in the area of each disease or risk factor of concern. They provided a best estimate and a "confidence" interval. These probability distributions were pooled, giving equal weighting to each distribution (i.e. to each expert reply), to obtain a combined probability distribution for the attributable fraction. Additional details on methods and results of this review have been published previously [2,30]. The resulting attributable fractions, specified by region/age group where applicable, have been combined with WHO disease statistics by country, gender and age group[19] to result into the estimate of the environmental burden of disease by country. The attributable environmental fractions used here are regional fractions, and not national as is the case for the indicators used in the first part of the country profile. The approach by disease rather than by risk factor avoids overlaps or double-counting.

Results are expressed in total number of deaths and DALYs per capita, and percentage of the national burden of disease attributable to the environment.

### (c) Environmental burden by disease category (Part 3)

Part 3 of the profile is a breakdown by disease group of the information given in Part 2, applying the same methods. It indicates the country's yearly number of DALYs per capita attributable to environmental factors by disease group.

For comparison, the world's lowest and highest country rates are provided for each disease group. The bar chart provides an indication of the country's situation for a particular disease group in comparison to other countries: A full bar indicates that the country's environmental DALYs per capita for the particular disease category is close to the highest rate encountered in any country, and a small bar indicates a rate close to the lowest encountered.

## Results

The country profile of environmental burden of disease for Brazil – overlaid by a reading guide – is provided in Figure 1 as an example. The profiles are available for 192 countries on the WHO web site [11]. They are structured in three parts, following the methods outlined above. In addition they provide some basic socio-economic and health indicators putting the data into context.

Additional file 1 provides a summary by country of the estimated number of deaths due to the three major risk factors unsafe 'water, sanitation & hygiene', 'indoor air pollution from solid fuel use' and 'outdoor air pollution', as well as basic exposure parameters. The last three columns of the Additional file 1 summarize estimates of the potentially preventable burden of the total environment, in premature deaths, DALYs per capita, and in percent of the country's total disease burden.

These results show that in 23 countries, the disease burden from the two risk factors 'unsafe water, sanitation & hygiene' and 'indoor air pollution from solid fuel use' alone accounts for more than one tenth of the country's total disease burden, amounting together to 3 million deaths globally. Outdoor air pollution adds another 860,000 deaths annually.

The disease prevention opportunities of environmental action per disease group differ generally by development status of a country. While contributions to infectious diseases, particularly diarrhoea and respiratory infections, are highest in the least developed countries, non-communicable diseases such as cancers and cardiovascular diseases are more affected in higher income countries.

**Figure 2:**
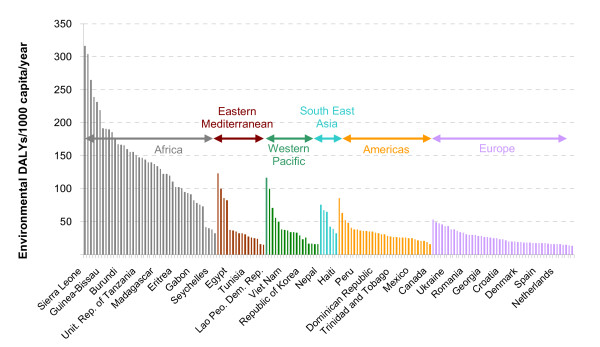
**Environmental DALYs per capita, by country, year 2002.** Country grouping corresponds to WHO Regions (WHO 2002).

The "environmental DALYs" per capita provide an overall measure of the environmental disease burden rate by country. They vary widely across countries, similar to the total burden of disease (Figure 2). Globally, 24% of the total disease burden, or 13 million premature deaths, could potentially be prevented through environmental improvements (or shifted to other causes of premature death or disability).

## Discussion

In 2007, WHO released the first ever country-by-country estimates of the impact environmental factors have on health for 192 Member states (WHO counts 193 Member states since 2006, but the latest health data available is from 2002, when only 192 countries were members of the organization) [11]. These country estimates are a first step to assisting national decision-makers in the sectors of health and environment to set priorities for preventive action. They can form an initial basis for orienting action to prevent disease by environmental improvements. Taken in a relative context, they can also be used as intra-national comparisons of broad environmental categories.

Strictly speaking, the disease burden that could be prevented through environmental action does not ensure that the concerned population would live a healthy life until standard life expectancy or beyond. It is likely that part of that population, in particular as life expectancy increases, is shifted to other causes of disease instead.

The number of environmental DALYs is an indication of the burden per capita that could be prevented by environmental improvement, and is a value that lends itself to inter-country comparison. This value is also the most meaningful one for capturing the disease burden attributable to the environment (next to last column in the Additional file 1), or the increase in healthy life years of a population when reducing environmental risks. The percentage of the total country's burden of disease that could be prevented by environmental improvements provides an indication of the total disease prevention that could be achieved within a country through environmental action.

Comparing the country rates of DALYs per capita by disease group with the world's lowest and highest country rates (comparison within the same line of the profile) provide an indication on the performance of the country with other countries. Values on the left ("lowest country rate") and on the right ("highest country rate") give an idea of the extreme values that were found within the 192 countries for which the exercise was done. Comparing however the country's DALYs per capita across disease groups (comparison across the "country rate" column of the profile) provides an indication of which diseases are the most affected by the environment in the country and points to the greatest prevention opportunities.

**Figure 3:**
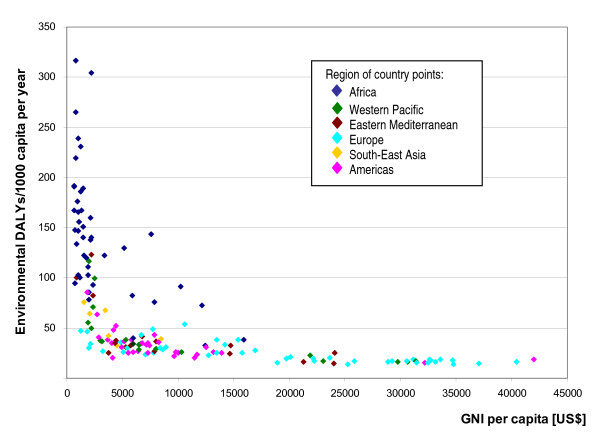
**Environmental burden (DALYs/capita) versus GNI (Gross National Income).** Country grouping corresponds to WHO Regions (WHO 2002).

**Figure 4:**
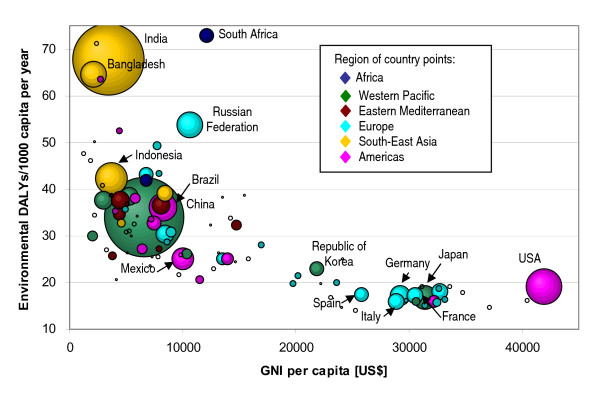
**Variability of environmental burden (DALY/capita) by GNI (Gross National Income).** Countries of EBD below 75 DALYs/1000 capita. Country grouping corresponds to WHO Regions (WHO 2002). Note: The surface of data points within the graph represents the country's population size; when considering trends, larger countries therefore appear more prominently.

Plotting the environmental DALYs per capita versus the GNI (Figure 3) shows a strong decrease in environmental burden as GNI increases, before stabilizing around 15 DALYs/1 000 capita. A closer look at the values below 75 DALYs per 1 000 capita (Figure 4) shows that countries may perform rather differently in terms of environmental health impacts for a similar GNI. For example, with a GNI between 5 000 and 10 000 US$ per capita, the environmental burden of disease can range between around 20 and 55 DALYs per capita, which is more than a 2.5-fold difference. Such differences seem to support that affordable environmental management options may significantly impact on population health.

For countries with GNI values below 5 000 US$, the environmental burden can even differ by a factor of five. In many of these low-income countries, the disease burden is characterized by a high child mortality which is often linked to insufficient health care (the case-fatality rates are high) and/or malnutrition. Recurrent infections of diarrhoea and pneumonia, and malaria caused by poor sanitary conditions, solid fuel use and poor vector management therefore put children at particular risk and impact heavily on a country's disease burden.

Low income countries suffer the most from environmental health factors, losing up to 20 times more healthy years of life per person per year than high income countries. However, the data show that even in countries with better environmental conditions almost one sixth of the disease burden could be prevented.

As much of the mortality from diarrhoea and respiratory infections concerns children under the age of five years in the lower income countries, it also becomes apparent that addressing unsafe water, sanitation & hygiene and indoor air pollution from solid fuel use can significantly reduce child mortality in many countries, for example by more than 25% in about 20 countries. Adding the malaria deaths in children that could be prevented through environmental management would increase this share even further.

In higher income countries, the environmental component of the disease burden operates mainly through non-communicable diseases, including cancers and cardiovascular diseases. These deaths occur at a relatively late stage in life, displaying relatively high premature death rates, but relatively low DALY rates (based on the years of life lost rather than the number of deaths).

Although we have not assessed the health impacts of social determinants (such as levels of poverty, education, employment conditions [31]), investments in these will also have a positive impact on health. These determinants act as modifiers of the environmental determinants. Wealthier people can often better protect themselves against environmental risks. For example, people with higher income can possibly afford bottled water, while the poor may not; poorer people often have more dangerous jobs, or live closer to contaminated environments. On the other hand, everyone is exposed to outdoor air pollution and other ubiquitous determinants.

The presented estimates of total disease burden caused by the environment provide an approximate estimate of burden that could potentially be prevented, given the availability and successful implementation of effective intervention. Due to the estimation by disease rather than by risk factor, issues such as interactions between risk factors or multicausality have not been addressed specifically.

The estimates may have the following main sources of uncertainty: (1) relevant exposure databases available for the majority of countries are limited; while survey data for access to safe water, basic sanitation and use of solid fuels are now available for most countries, the missing data have been modelled; (2) given the lack of exposure data, the number of risks for which disease burden could be estimated with exposure-based methods was limited; the health impacts specific to other environmental risks could not be estimated on a country-by-country basis; (3) comprehensive health statistics are lacking for many countries; WHO estimates rely on modelling when data are incomplete; they however constitute the only comprehensive and consistent database of this type; (4) applying regional attributable fractions to country health statistics may not fully reflect the country-specific environmental exposures, in particular if these differ significantly from the regional mean; for example, if the risks for respiratory infections (e.g. solid fuel use, outdoor air pollutions, second-hand smoke) in a specific country differ significantly from the regional mean, then the presented estimates will carry larger uncertainties; provided however that the country's environmental situation is relatively similar to the one prevailing in the region (e.g. similar environmental policies, proportion of urban population, industrial activities), then the presented estimates may provide useful indications; (5) within-country variations in health status and exposure: if vulnerable populations are more likely to be exposed (which often applies with environmental exposures), the method may lead to an underestimation of effects.

The magnitude of uncertainties is difficult to evaluate. While in principle it would be possible to estimate uncertainties of the different components of the estimate, i.e. uncertainties in health data, the attributable fractions and the exposure data, it is more difficult to estimate the error generated by the use of regional attributable fractions as opposed to national estimates. In the absence of quantified information on uncertainties around these components of the estimate, a quantitative estimate of a "confidence interval" is not appropriate. For improving accuracy and reducing uncertainties, a more refined estimate based on national data may be carried out.

## Conclusion

The comparative estimates of the proportion of disease burden attributable to the environment vary between 13 and 37% according to the country. These estimates show that the potential for improving health through a healthier environment is substantial in literally every country around the world. Environmental modifications could particularly reduce child mortality in many of the lowest income countries, but also play an important role in preventing non-communicable diseases in the higher income countries.

We acknowledge that the estimates proposed in this publication carry quite large uncertainties, and should primarily serve as preliminary estimates to refine at country level with additional input data. They do however, for the first time, provide an overview of the disease prevention opportunities by disease group (which have already been grossly linked to intervention area [2], and on which additional work is in preparation) through environmental action for 192 countries. Additional exposure-based methods for environmental risks are currently being developed, and exposures assessed, which could improve the accuracy of future estimates. Ideally, such estimates could also be extended to cover risk factors from other areas (such as physical inactivity, malnutrition, smoking, poverty etc.). Such a "health determinants picture" would then provide a more complete picture of a country's prevention opportunities. Basic methods for the assessment of health impacts from many of such risks are already available [1,14].

## Competing interests

The author(s) declare that they have no competing interests.

## Authors' contributions

APU and CC developed the concept of the country profiles. APU performed most of the data analyses and drafted the manuscript. SB developed the burden of disease estimates for outdoor air pollution and indoor smoke from solid fuel use. All authors read and approved the final manuscript.

## Disclaimer

The authors are staff members of the World Health Organization. The authors alone are responsible for the views expressed in this publication and they do not necessarily represent the decisions or the stated policy of the World Health Organization.

## Supplementary Material

Additional file 1 Disease burden attributable to the environment, and selected exposures, by country, year 2002. Disease burden for the risk factors 'water, sanitation and hygiene', 'indoor air pollution' and 'outdoor air pollution', and for the total environment. Click here for file
