# Supplementary material for: The impact of the environment on health by country: a meta-synthesis
Source: Environ Health. 2008 Feb 25;7:7. doi: 10.1186/1476-069X-7-7 (PMC2276491; doi:10.1186/1476-069X-7-7)
Supplement: Additional file 1 — Disease burden attributable to the environment, and selected exposures, by country, year 2002. Disease burden for the risk factors 'water, sanitation and hygiene', 'indoor air pollution' and 'outdoor air pollution', and for the total environment. [file 1476-069X-7-7-S1.pdf]

Table 1: Disease burden attributable to the environment, and selected exposures, by country, year 2002[§,†]

		Water, sanitation & hygiene			Indoor air pollution		Outdoor air pollution			Total environment		
Country	Population ('000)	Improved water (%)	Improved sanitation (%)	Deaths per year[‡]	Population using solid fuel (%)	Deaths per year	Annual PM10 [µg/m³]	Urban population (%)#	Deaths per year	Percent of total burden (%)	Environmental DALYs/ 1000 capita	Environmental deaths
Afghanistan	*22 930*	39	34	36 800	>95	23 900	27	16	400	29%	217	144 700
Albania	*3 141*	96	91	300	50	<100	58	9	200	19%	30	4 400
Algeria	*31 266*	85	92	7 000	<5	400	65	25	1 900	24%	42	42 700
Andorra	*69*	100	100	-	<5	-	41	39	-	14%	18	100
Angola	*13 184*	53	31	43 500	>95	22 000	113	20	1 800	37%	304	116 200
Antigua & Barbuda	*73*	91	95	-	46	-	26	33	-	13%	23	100
Argentina	*37 981*	96	91	400	<5	-	78	74	12 200	15%	25	50 100
Armenia	*3 072*	92	83	<100	26	100	84	54	1 600	16%	26	4 700
Australia	*19 544*	100	100	-	<5	-	18	72	700	14%	16	21 700
Austria	*8 111*	100	100	-	<5	-	32	37	1 100	14%	16	11 400
Azerbaijan	*8 297*	77	54	800	49	1 800	64	23	1 400	19%	36	12 900
Bahamas	*310*	97	100	-	<5	-	18	72	-	15%	26	300
Bahrain	*709*	na	na	-	<5	-	65	26	<100	14%	16	400
Bangladesh	*143 809*	74	39	60 300	89	46 000	157	9	8 200	25%	65	300 900
Barbados	*269*	100	100	-	<5	-	95	4	-	13%	22	300
Belarus	*9 940*	100	84	-	19	200	9	49	-	20%	43	29 700
Belgium	*10 296*	100	100	-	<5	-	41	34	2 000	14%	19	17 000
Belize	*251*	91	47	-	43	-	12	25	-	19%	35	300
Benin	*6 558*	67	33	6 500	95	6 300	51	19	300	34%	156	29 300
Bhutan	*2 190*	62	70	1 200	no data	-	13	3	-	25%	72	5 600
Bolivia	*8 645*	85	46	3 700	34	1 200	72	45	1 000	23%	63	17 400
Bosnia-Herzegovina	*4 126*	97	95	-	50	<100	22	37	300	16%	26	6 200
Botswana	*1 770*	95	42	300	65	200	25	13	<10	13%	91	5 000
Brazil	*176 257*	90	75	15 000	13	4 100	35	45	12 900	17%	36	228 000
Brunei Darussalam	*350*	na	na	-	no data	-	48	30	-	14%	17	200
Bulgaria	*7 965*	99	99	-	17	<100	59	37	3 400	16%	29	18 500
Burkina Faso	*12 624*	61	13	19 500	>95	21 500	97	14	1 100	34%	231	83 100
Burundi	*6 602*	79	36	8,200	>95	6 600	99	7	200	27%	167	32 000
Cambodia	*13 810*	41	17	10 600	>95	1 600	51	8	200	26%	100	41 600
Cameroon	*15 729*	66	51	12 700	83	12 900	86	27	2 000	28%	138	64 500
Canada	*31 271*	100	100	-	<5	-	21	79	2 700	13%	16	36 500
Cape Verde	*454*	80	43	<100	36	<100	33	5	<10	23%	40	600
Central African Rep.	*3 819*	75	27	4 200	>95	2 900	24	20	<100	25%	156	18 500
Chad	*8 348*	42	9	10 200	>95	8 700	73	19	600	31%	190	45 900
Chile	*15 613*	95	91	200	<5	-	62	57	2 300	15%	21	14 800
China	*1 302 307*	77	44	95 600	80	380 700	80	37	275 600	22%	34	2 346 900
Colombia	*43 526*	93	86	2 300	20	1 900	42	41	2 700	17%	33	46 300
Comoros	*747*	86	33	300	77	100	125	7	<100	29%	78	1 600
Congo	*3 633*	58	27	1 400	85	700	74	54	500	23%	95	10 100
Cook Islands	*18*	94	100	-	no data	-	no data	na	-	19%	31	<100
Costa Rica	*4 094*	97	92	100	23	100	40	46	200	16%	22	3 200
Croatia	*4 439*	100	100	-	12	-	35	32	900	14%	23	8 400
Cuba	*11 271*	91	98	200	21	100	38	39	1 600	16%	23	15 300
Cyprus	*796*	100	100	-	<5	-	60	48	300	13%	17	1 400
Czech Republic	*10 246*	100	98	-	<5	-	42	24	1 700	15%	21	17 600
Côte d'Ivoire	*16 365*	84	37	14 700	74	9 300	38	26	700	27%	140	68 500
Dem. Peo. Rep. Korea	*22 541*	100	59	2 800	no data	-	88	31	4 900	20%	43	45 500
Dem. Rep. Congo	*51 201*	46	30	100 300	>95	47 100	57	22	2 900	33%	219	324 900
Denmark	*5 351*	100	100	-	<5	-	24	38	600	14%	19	9 200
Djibouti	*693*	73	82	700	5	<100	68	76	200	30%	123	2 400
Dominica	*78*	97	84	-	21	-	34	22	-	15%	26	100
Dominican Republic	*8 616*	95	78	1 100	15	<100	36	43	500	17%	34	10 200
Ecuador	*12 810*	94	89	1 700	<5	100	34	48	500	19%	35	15 100
Egypt	*70 507*	98	70	11 400	<5	700	136	32	15 000	19%	38	96 800
El Salvador	*6 415*	84	62	800	33	400	48	27	300	18%	37	8 000
Equatorial Guinea	*481*	43	53	400	no data	-	12	25	-	27%	144	2 000
Eritrea	*3 991*	60	9	2 300	80	2 800	109	16	200	28%	103	11 500
Estonia	*1 338*	100	97	-	16	-	19	37	100	20%	39	3 700
Ethiopia	*68 961*	22	13	57 000	>95	56 700	88	7	2 000	28%	147	299 400
Fiji	*831*	47	72	<100	40	<100	17	25	-	17%	34	1 100
Finland	*5 197*	100	100	-	<5	-	16	42	200	15%	19	8 200
France	*59 850*	100	na	-	<5	-	25	42	4 800	14%	17	80 100

		Water, sanitation & hygiene			Indoor air pollution		Outdoor air pollution			Total environment		
Country	Population ('000)	Improved water (%)	Improved sanitation (%)	Deaths per year‡	Population using solid fuel (%)	Deaths per year	Annual PM10 [µg/m³]	Urban population (%)#	Deaths per year	Percent of total burden (%)	Environmental DALYs/ 1000 capita	Environmental deaths
Gabon	*1 306*	88	36	400	28	200	13	45	-	23%	83	3 400
Gambia	*1 388*	82	53	800	>95	600	138	19	200	30%	111	4 700
Georgia	*5 177*	82	94	-	43	100	46	40	2 200	16%	27	10 900
Germany	*82 414*	100	100	-	<5	-	29	32	10 400	14%	17	132 200
Ghana	*20 471*	75	18	8 600	87	5 600	42	20	600	27%	93	55 600
Greece	*10 970*	na	na	-	<5	-	34	46	2 800	16%	20	20 000
Grenada	*80*	95	96	-	48	-	49	na	-	16%	35	100
Guatemala	*12 036*	95	86	2 900	62	3 300	60	23	400	22%	52	20 700
Guinea	*8 359*	50	18	8 500	>95	800	63	22	400	29%	140	33 600
Guinea-Bissau	*1 449*	59	35	1 400	95	1 200	84	20	100	31%	192	8 200
Guyana	*764*	83	70	300	59	<100	13	28	-	18%	48	1 300
Haiti	*8 218*	54	30	5 000	>95	2 900	47	31	500	20%	86	23 400
Honduras	*6 781*	87	69	1 500	57	500	69	31	500	19%	41	8 100
Hungary	*9 923*	99	95	-	<5	-	34	32	1 900	16%	28	21 700
Iceland	*287*	100	100	-	<5	-	21	59	<100	14%	14	300
India	*1 049 550*	86	33	402 200	82	407 100	84	19	120 600	24%	68	2 621 100
Indonesia	*217 131*	77	55	31 200	72	15 300	114	22	28 800	20%	42	356 100
Iran	*68 070*	94	na	na	<5	200	68	42	9 000	20%	37	81 200
Iraq	*24 510*	81	79	12 200	<5	600	167	58	7 500	26%	88	58 600
Ireland	*3 911*	na	na	-	<5	-	15	32	<100	14%	18	5 300
Israel	*6 304*	100	na	na	<5	-	53	80	1 500	13%	14	5 600
Italy	*57 482*	na	na	-	<5	-	37	27	8 400	14%	16	90 800
Jamaica	*2 627*	93	80	100	45	<100	43	27	200	14%	21	3 000
Japan	*127 478*	100	100	-	<5	-	33	64	23 800	15%	15	174 800
Jordan	*5 329*	97	93	600	<5	-	69	49	500	20%	31	5 200
Kazakhstan	*15 469*	86	72	300	<5	<100	25	43	2 300	20%	49	39 300
Kenya	*31 540*	61	43	21 800	63	13 000	38	16	600	24%	101	93 900
Kiribati	*87*	65	40	-	no data	-	no data	na	-	16%	45	100
Kuwait	*2 443*	na	na	-	<5	-	129	74	300	14%	15	800
Kyrgyzstan	*5 067*	77	59	600	76	1 600	36	16	400	21%	46	9 700
Lao Peo. Dem. Rep.	*5 529*	51	30	4 900	>95	2 400	25	4	<100	29%	117	20 300
Latvia	*2 329*	99	78	-	10	-	17	42	<100	18%	38	6 500
Lebanon	*3 596*	100	98	200	<5	-	43	74	600	18%	32	4 500
Lesotho	*1 800*	79	37	1 300	83	400	94	10	<100	16%	122	7 500
Liberia	*3 239*	61	27	4 300	no data	-	39	17	<100	30%	231	21 300
Libyan Arab Jamahiriya	*5 445*	na	97	na	<5	<100	121	85	1 600	17%	25	4 500
Lithuania	*3 465*	na	na	-	<5	-	29	42	700	19%	34	8 300
Luxembourg	*447*	100	100	-	<5	-	17	19	-	15%	18	600
Madagascar	*16 916*	46	32	16 900	>95	11 700	51	15	600	32%	134	65 600
Malawi	*11 871*	73	61	17 100	>95	13 300	88	10	600	27%	191	68 400
Malaysia	*23 965*	99	94	300	<5	<100	28	28	500	18%	26	25 600
Maldives	*309*	83	59	<100	no data	-	54	27	<100	21%	41	500
Mali	*12 623*	50	46	20 200	>95	16 900	102	11	700	35%	239	85 400
Malta	*393*	100	na	-	<5	-	no data	na	-	14%	16	500
Marshall Islands	*52*	87	82	-	no data	-	no data	na	-	21%	51	100
Mauritania	*2 807*	53	34	3 200	56	2 300	42	34	200	33%	160	13 200
Mauritius	*1 210*	100	94	-	<5	-	47	12	<100	18%	32	1 300
Mexico	*101 965*	97	79	4 800	14	2 400	49	56	7 200	17%	25	80 300
Micronesia	*108*	94	28	<100	no data	-	no data	na	-	24%	48	200
Monaco	*34*	100	100	-	<5	-	no data	na	-	14%	15	<100
Mongolia	*2 559*	62	59	800	51	300	16	29	-	22%	50	4 400
Morocco	*30 072*	81	73	6 000	5	600	27	37	700	20%	35	32 300
Mozambique	*18 537*	43	32	26 900	80	9 700	44	25	900	28%	186	108 500
Myanmar	*48 852*	78	77	21 700	>95	14 700	75	14	3 900	25%	73	132 300
Namibia	*1 961*	87	25	700	65	200	50	12	<100	17%	76	4 700
Nauru	*13*	na	na	-	no data	-	no data	na	-	18%	43	<100
Nepal	*24 609*	90	35	14 700	81	7 500	161	4	700	25%	76	62 900
Netherlands	*16 067*	100	100	-	<5	-	38	52	3 600	14%	16	21 800
New Zealand	*3 846*	na	na	-	<5	-	16	70	<100	14%	17	4 500
Nicaragua	*5 335*	79	47	1 200	64	700	32	26	<100	22%	39	5 800
Niger	*11 544*	46	13	22 700	>95	13 600	86	10	500	34%	265	86 400
Nigeria	*120 911*	48	44	119 700	67	79 000	95	27	14 700	29%	166	581 400
Niue	*2*	100	100	-	no data	-	no data	na	-	19%	34	<100
Norway	*4 514*	100	100	-	<5	-	22	33	400	14%	16	7 500
Oman	*2 768*	na	na	-	<5	-	124	36	300	19%	25	1 600
Pakistan	*149 911*	91	59	103 300	81	70 700	165	27	28 700	28%	82	402 200

		Water, sanitation & hygiene			Indoor air pollution		Outdoor air pollution			Total environment		
Country	Population ('000)	Improved water (%)	Improved sanitation (%)	Deaths per year‡	Population using solid fuel (%)	Deaths per year	Annual PM10 [$\mu g/m^3$]	Urban population (%)#	Deaths per year	Percent of total burden (%)	Environmental DALYs/ 1000 capita	Environmental deaths
Palau	*20*	85	83	-	no data	-	no data	na	-	18%	33	<100
Panama	*3 064*	90	73	200	33	<100	58	29	200	17%	26	2 600
Papua New Guinea	*5 586*	39	44	2 100	90	1 600	11	6	-	25%	71	12 400
Paraguay	*5 740*	86	80	700	53	300	103	25	400	17%	31	5 300
Peru	*26 767*	83	63	3 900	33	1 500	62	53	3 100	20%	38	37 100
Philippines	*78 580*	85	72	10 600	45	6 900	34	41	3 900	20%	39	102 100
Poland	*38 622*	na	na	-	<5	-	40	32	6 000	17%	25	66 100
Portugal	*10 049*	na	na	-	<5	-	27	56	1 900	14%	20	15 400
Qatar	*601*	100	100	-	<5	-	57	65	<100	16%	18	200
Republic of Korea	*47 430*	92	na	na	<5	-	43	74	6 800	20%	26	54 500
Republic of Moldova	*4 270*	92	68	100	63	200	41	27	900	17%	34	9 000
Romania	*22 387*	57	na	na	23	300	76	33	9 400	17%	31	46 900
Russian Federation	*144 082*	97	87	700	9	400	25	48	37 200	20%	54	493 100
Rwanda	*8 272*	74	42	11 700	>95	8 100	100	6	200	31%	167	40 400
Saint Kitts and Nevis	*42*	99	95	-	<5	-	33	29	-	16%	31	100
Saint Lucia	*148*	98	89		63	-	74	na	-	16%	26	100
Saint Vincent & Gren.	*119*	na	na	-	31	-	56	19	-	15%	28	100
Samoa	*176*	88	100	-	70	-	no data	na	-	20%	33	200
San Marino	*27*	na	na	-	<5	-	19	na	-	15%	16	<100
Sao Tome & Principe	*157*	79	25	<100	95	<100	76	27	-	27%	73	400
Saudi Arabia	*23 520*	na	na	na	<5	-	91	40	2 400	20%	32	20 700
Senegal	*9 855*	76	57	6 500	53	5 400	93	29	900	31%	120	32 300
Serbia-Montenegro	*10 535*	93	87	-	no data	-	17	20	100	15%	27	21 000
Seychelles	*80*	88	na	-	<5	-	no data	na	-	19%	38	100
Sierra Leone	*4 764*	57	39	11 500	92	7 600	69	20	400	33%	316	44 300
Singapore	*4 183*	100	100	-	<5	-	48	79	1 000	13%	14	3 200
Slovakia	*5 398*	100	99	-	<5	-	31	17	400	16%	25	9 300
Slovenia	*1 986*	na	na	-	8	-	44	21	300	14%	20	2 900
Solomon Islands	*463*	70	31	200	95	<100	16	na	-	24%	56	800
Somalia	*9 480*	29	26	14 000	no data	-	35	20	300	27%	184	47 800
South Africa	*44 759*	88	65	11 900	18	1 000	24	43	1 000	16%	73	109 100
Spain	*40 977*	100	100	-	<5	-	30	42	5 800	14%	17	58 500
Sri Lanka	*18 910*	79	91	600	67	3 100	93	8	1 000	18%	33	29 500
Sudan	*32 878*	70	34	17 200	>95	4 400	219	17	3 700	24%	86	78 000
Suriname	*432*	92	94	<100	no data	-	13	55	-	19%	38	600
Swaziland	*1 069*	62	48	500	64	400	71	7	<100	17%	130	4 400
Sweden	*8 867*	100	100	-	<5	-	19	30	500	14%	15	14 500
Switzerland	*7 171*	100	100	-	<5	-	27	42	800	13%	15	9 500
Syrian Arab Republic	*17 381*	93	90	1 800	32	400	89	38	1 600	17%	26	13 300
Tajikistan	*6 195*	59	51	1 800	75	1 600	57	11	500	21%	47	12 000
Thailand	*62 193*	99	99	2 800	72	4 600	77	16	2 800	19%	39	86 100
TFYR of Macedonia	*2 046*	na	na	na	30	-	29	29	200	15%	24	3 100
Timor-Leste	*739*	58	36	100	no data	-	no data	na	-	30%	61	1 800
Togo	*4 801*	52	35	2 600	87	4 100	45	16	200	28%	122	16 900
Tonga	*103*	100	96	-	56	-	no data	na	-	19%	30	100
Trinidad and Tobago	*1 298*	91	100	-	8	-	22	4	<10	13%	26	1 700
Tunisia	*9 728*	93	85	600	<5	100	46	30	700	17%	27	10 300
Turkey	*70 318*	96	88	6 000	11	2 500	56	61	18 800	19%	30	86 700
Turkmenistan	*4 794*	72	62	1 000	<5	-	73	18	700	22%	49	9 100
Tuvalu	*10*	93	90	-	no data	-	no data	na	-	22%	61	<100
Uganda	*25 004*	60	43	26 800	>95	19 900	33	5	100	28%	151	106 900
Ukraine	*48 902*	96	96	<100	6	200	29	42	15 200	19%	43	155 200
United Arab Emirates	*2 937*	100	98	<100	<5	-	109	70	400	18%	25	2 000
United Kingdom	*59 068*	100	na	-	<5	-	26	53	12 400	14%	18	101 300
Unit. Rep. of Tanzania	*36 276*	62	47	28 200	>95	27 500	38	21	1 000	26%	148	154 800
US of America	*291 038*	100	100	-	<5	-	24	72	41 200	13%	19	397 800
Uruguay	*3 391*	100	100	<100	<5	-	154	44	1 300	16%	26	5 800
Uzbekistan	*25 705*	82	67	500	72	5 300	81	21	4 300	18%	30	33 500
Vanuatu	*207*	60	50	<100	79	-	10	14	-	20%	37	300
Venezuela	*25 226*	83	68	1 400	5	100	16	62	-	17%	27	22 000
Viet Nam	*80 278*	85	61	9 400	70	10 600	66	21	6 300	23%	38	125 600
Yemen	*19 315*	67	43	16 900	42	7 000	82	15	1 000	28%	100	51 300
Zambia	*10 698*	58	55	13 700	87	8 600	71	29	1 100	25%	176	57 900
Zimbabwe	*12 835*	81	53	5 400	72	1 900	43	26	600	15%	102	44 200
WORLD	*6 213 869*	78	49	1 635 200	52	1 497 000	61	34	865 000	24%	55	13 475 900

[§] Additional information on data sources can be found at
http://www.who.int/quantifying_ehimpacts/countryprofilesexplanatoryandsources.pdf
[†] 2002 is the most recent year of health data available from WHO
[‡] Only diarrhoea deaths
[#] Population living in cities of more than 100 000 inhabitants
